# A Brief Up-Date of the Use of Sodium Oxybate for the Treatment of Alcohol Use Disorder

**DOI:** 10.3390/ijerph13030290

**Published:** 2016-03-05

**Authors:** Fabio Caputo, Teo Vignoli, Claudia Tarli, Marco Domenicali, Giorgio Zoli, Mauro Bernardi, Giovanni Addolorato

**Affiliations:** 1 Department of Internal Medicine, SS Annunziata Hospital, Cento, Ferrara 44042, Italy; g.zoli@ausl.fe.it; 2 “G. Fontana” Centre for the Study and Multidisciplinary Treatment of Alcohol Addiction, Department of Clinical Medicine, University of Bologna, Bologna 40130, Italy; m.domenicali@unibo.it (M.D.); mauro.bernardi@unibo.it (M.B.); 3 Unit for Addiction Treatment, Department of Mental Health, Lugo, Ravenna 48022, Italy; teo.vignoli@ausl.ra.it; 4 Alcohol Use Disorders Unit, Department of Internal Medicine, Catholic University of Rome, Rome 00168, Italy; claudia.tarli@gmail.com (C.T.); giovanni.addolorato@unicatt.it (G.A.)

**Keywords:** alcohol use disorder, pharmacological treatment, sodium oxybate

## Abstract

The treatment of alcohol use disorder (AUD) with sodium oxybate (SMO) or gamma-hydroxybutyric acid (GHB) was introduced in Italy and Austria more than 20 years and 15 years ago, respectively, and it is now widely employed to treat alcohol withdrawal syndrome (AWS) and to maintain alcohol abstinence. These indications derive from its similar structure to the inhibitory neurotransmitter γ-amino-butyric acid (GABA), exerting an ethanol-mimicking effect, because it binds to GABA_B_ receptors. Craving for, and abuse of, SMO remain a controversial issue; even though these unfavorable effects are evident in poly-drug addicted patients and in those with psychiatric diagnosis of borderline personality disorder. In addition, despite cases of severe intoxication and deaths being widely documented when GHB is used as “street drug”; its clinical use remains safe. Thus, the aim of the present review is to examine the role of SMO in the treatment of AUD, its possible implications in reducing alcohol consumption, and cases of abuse, and severe intoxication due to SMO during its clinical use in the treatment of AUD.

## 1. Introduction

Sodium oxybate (SMO) (gamma-hydroxybutyric acid (GHB), gamma hydroxybutyrate, or sodium salt of GHB), is a short-chain fatty acid that occurs naturally in the mammalian brain, in particular in the thalamus, hypothalamus, and basal ganglia [1]. SMO is structurally similar to the inhibitory neurotransmitter γ-amino-butyric acid (GABA) binding to GABA_B_ receptors [1,2,3]. Its functions are as both a precursor and a metabolite of the GABA system. Irrespective of the brain SMO concentration, it is far from certain that SMO interacts directly with the GABA_A_ receptor [4]. SMO is primarily eliminated by the liver by the enzyme GHB dehydrogenase, and by a still not fully ascertained process of beta-oxidation [1]. Only a modest quantity of SMO remains unmodified (2%–5%) and eliminated with urine with a relatively short window of detection (3–12 h) [2]. After exogenous ingestion of SMO, its maximum concentration occurs after 20–40 min, and its half-life is 30–50 min [5]. It is thought that the alcohol-mimicking effect of SMO is related to the effects of the dopamine increase mediated by GABA_B_ receptors in the mesocorticolimbic circuitry [1,3,6]. This results in a decreasing GABA release with a consequent increase of dopamine within this system [1]. Indeed, the exact mechanism by which GABA_B_ agonists exert their effect on alcohol craving is still a matter of research [7]. Most evidence suggests that mesolimbic dopaminergic neurons, originating in the ventral tegmental area and projecting their neurons into the nucleus accumbens, could play a pivotal role in the regulation of alcohol craving, being stimulated by alcohol consumption [7].

In the United States, the Food and Drug Administration approved SMO (Xyrem^®^) as a Schedule III Controlled Substance to treat cataplexy and excessive daytime sleepiness in patients with narcolepsy [8,9]. In Europe, SMO is used in Germany (Somsanit^®^) and in France (Gamma-OH^®^) for intravenous anesthesia, and has been used for the treatment of alcohol use disorder (AUD) in Italy (Alcover^®^) since 1992, and in Austria since 1999. Indeed, several studies have demonstrated that SMO is a safe and effective drug for treating AUD.

## 2. SMO for the Treatment of Alcohol Withdrawal Syndrome

Alcohol withdrawal syndrome (AWS) represents a clinical condition that usually develops in alcohol-dependent patients within 6–24 h after the abrupt discontinuation or decrease of alcohol consumption [10]. SMO was tested in preclinical and clinical settings for the treatment of AWS [11]. A meta-analysis performed in 2010 by the Cochrane Collaboration showed that SMO (50–100 mg/kg/day) is more effective than placebo in reducing the CIWA-Ar score with an equal efficacy to benzodiazepines and clomethiazole without any differences in the onset of side effects [12]. Recently, the GATE 1 study (phase IV, multicenter, multinational, randomized, double-blind, double-dummy, with parallel groups) showed that SMO presents a similar efficacy to oxazepam (one of the gold standard benzodiazepines) in the treatment of uncomplicated AWS [13].

## 3. SMO as an Anti-Craving Drug to Treat Alcohol Use Disorder

Thanks to the ability of SMO to inhibit voluntary ethanol consumption, SMO is used for the treatment of AUD with very encouraging results in maintaining total alcohol abstinence.In particular, almost 50%–60% of treated patients achieve and maintain alcohol abstinence at the end of the first three months of treatment [14]; in addition, SMO is at least as effective as naltrexone (NTX) or disulfiram (DF) in the maintenance of abstinence in alcohol-dependent patients [12]. Concerns have been raised about the risk of developing addiction to, misuse, or abuse of SMO. However, clinical trials have shown that episodes of craving for, and abuse of, SMO in alcoholics are very limited (~10% of cases), and are mainly confined to patients with AUD associated with poly-drug addiction and psychiatric co-morbidity [3,11,12,15]. Moreover, cases of death related to SMO abuse have not been documented in clinical trials dedicated to the treatment of alcohol dependence [3,11] or narcolepsy [16]. However, “real-life trials” which would have improved the awareness of the negative impact of this phenomenon during the daily treatment of alcoholic patients with SMO have not been performed.

In addition, as reported recently by a survey among fifty Italian alcohologists [14], a consensus has been reached to prolong the treatment with SMO until a significant improvement in the motivation to abstain is achieved, to increase the dosages of SMO until the craving for alcohol is suppressed, and to not consider the treatment of SMO as being the “last chance” drug when no result has been achieved with other pharmacological and non-pharmacological treatments. From 50 to 100 mg/kg/day, fractioned into three to six daily administrations, could be considered a safe approach to use SMO [11,12,15]. About 30% of alcohol-dependent patients treated with SMO can develop side effects, represented by dizziness, sedation, and asthenia. These events do not, in general, require discontinuation of treatment, because the dizziness disappears spontaneously after the first doses, while sedation and asthenia disappear within 2–3 weeks. In addition, no side effects due to the combination of SMO 50 mg/kg and alcohol were observed in those SMO-treated alcoholics who were still drinking during the treatment [11,12,15]. This was also confirmed by a recent randomized, double-blind, double-dummy, cross-over trial in healthy volunteers aimed at exploring the pharmaco-dynamic interaction of the solid immediate release formulation of SMO and alcohol, which clearly showed that SMO and alcohol have separate adverse effect profiles, and that the objective effects of SMO are much less marked than those of alcohol, without any deleterious interaction [17]. However, larger clinical trials with alcohol dependent patients aimed at investigating a possible negative interaction during the concurrent intake of alcohol and SMO have not been performed, so that the suggestion not to drink during SMO administration represents a crucial message for physicians before prescribing this medication for AUD.

SMO has also been evaluated in combination with other drugs. In particular, an open, randomized, comparative study evaluated the efficacy of SMO in combination with NTX in maintaining alcohol abstinence compared to SMO and NTX used singly [18]. These data confirm that the two drugs combine their different actions synergistically without suppressing the favorable effects of each other. In SMO treatment-resistant chronic alcoholics (30%–40%), the combination with DF was proposed. SMO-DF combines the adverse effect of DF with the anti-reward effect of SMO [19].

## 4. Possible Role of SMO in Reducing Alcohol Consumption

The major outcome in the treatment of AUD remains total alcohol abstinence; this is because alcohol intake, even in low doses, continues to maintain the craving for alcohol and may also lead to abuse of alcohol [20]. Nevertheless, a reduction in alcohol intake, in particular for heavy drinkers, does lead to an improvement in physical and psychological health even if the patient does not achieve abstinence [21,22]. Moreover, a reduction of alcohol intake can represent a temporary step toward the achievement of complete abstinence [23,24]. In the light of these considerations, it has been demonstrated that all clinical studies testing the efficacy of SMO in maintaining abstinence from alcohol have recorded a number of patients who failed to achieve this primary end-point, but did reduce their alcohol intake [12]. In particular, SMO was shown to be significantly more effective than placebo in reducing the number of daily drinks (*p* < 0.00001) and in reducing relapses into heavy drinking (*p* < 0.00032) in a controlled clinical trial [25]. In a two-phase trial exploring the efficacy of dose-fractioning of SMO treatment, 17.4% of patients did not achieve complete abstinence but they significantly reduced their daily drinking (*p* < 0.05) at the end of the first three month phase [26]. An open multicenter study found a reduction of biomarkers of alcohol abuse after SMO treatment, and the group of patients who did not achieve complete abstinence, did reduce their average alcohol intake [27]. Maremmani *et al.* described a long term treatment with SMO in a population of treatment-resistant patients [28]; although the group was not so numerous, the partial responder group who reduced their alcohol intake for an average of 40% was larger than the total responders who achieved complete abstinence from alcohol (14.3% *vs.* 11.4%). More recent studies [29,30] investigating the efficacy of SMO in some particular population types (according to Lesch typology and with or without psychiatric co-morbidity) found that a relevant number of patients did not achieve complete abstinence, but they did not return to heavy drinking (41.7% of the total population) [29,30]. A recently published SMO trial [11] considered the cumulative days of abstinence (CAD) as the primary endpoint. This study demonstrated a borderline significance in favor of SMO in reducing CAD [11]. Considering SMO as a possible treatment option for reducing alcohol consumption, concerns remain about the side effects of the combination with alcohol.No additional sedative effects induced by drinking during SMO treatment were found in healthy subjects [17]. Moreover, considering that SMO is utilized both for treating AWS and to maintain abstinence, according to clinical practice, then complete abstinence from alcohol should not be considered an exclusion criterion for treatment with SMO [14].

In the light of these considerations, there is some preliminary evidence that SMO can be efficient in reducing alcohol intake in patients who fail to maintain total alcohol abstinence. It also seems that, for less motivated patients to achieve total alcohol abstinence immediately, the reduction in alcohol intake could be the primary end-point of SMO treatment, suggesting a role in harm reduction treatment. Unfortunately, the end points defined by published trials are varied and non-comparable: heavy drinking days, daily alcohol intake, total amount of alcohol intake, or cumulative days of abstinence. Thus, more studies to confirm these data and to explore the efficacy of SMO in patients considering alcohol reduction as their primary goal are warranted.

## 5. Severe Intoxications Due to GHB: These Do not Refer to Its Clinical Use

GHB as a “street drug”, sold for recreational use, is mostly reported in Anglo-Saxon countries [31]; however, experiences of this have also been reported in Italy [32,33]. “Street use” represents the first cause of GHB-related death [31]. Risk factors are unknown: dose/concentration, frequently combined use with other drugs, difficulties with dose titration [34], and narrow safety margins between a recreational dose and a fatal dose [35]. Cardiorespiratory depression is a documented dose-related effect of GHB [36], and it is likely to be the principal cause of death in GHB overdoses [31]. Whereas it is well known that a GHB blood concentration of 500 mg/L causes death due to cardiorespiratory depression, it is impossible to clearly define a “lethal” dose [4]. Reduced vigilance leading to trauma and driving impairment are other possible causes of GHB-related death [37]. Furthermore, considering that blood GHB levels increase substantially *post mortem*, even in persons in whom the cause of death excluded any possible exposure to GHB [38,39], a strictly causal relationship between GHB use and death is, sometimes, very difficult to clearly ascertain [4]. With regard to SMO treatment for AUD (Alcover^®^), there are no published data about related deaths [4]. In this regard, interestingly, only one case has been documented [40]; however, this was a heroin-dependent patient and the concomitant use of morphine would have played a crucial role as a cause of death.

## 6. Conclusions

According to many studies conducted to evaluate the pharmacological effects, SMO is efficient in the treatment of AWS and in achieving and maintaining total abstinence from alcohol.The pharmacological activity of this drug is well-known: thanks to its ethanol-mimicking effect binding GABA_B_ receptors, SMO increases dopamine levels in the mesocorticolimbic circuitry and determines a craving reduction. None of the above-mentioned trials reported serious side effects during the treatment with SMO; craving for, and abuse of, SMO in alcohol-dependent patients represent a very limited phenomenon (~10%–15% of cases); particular caution in prescribing this drug in the case of alcoholics with poly-drug addiction and/or a borderline personality disorder is warranted. In addition, due to its very short half-life (30–50 min), the fractioning of the SMO doses from three to six daily administrations avoids the occurrence of further sedative effects in patients who voluntarily use alcohol during the treatment with this drug [3,11,14,15]. Moreover, since clinical trials aimed at investigating the efficacy of SMO in reducing alcohol consumption have not been performed, and since concerns remain about the side effects of the combination with alcohol, it is hard to prescribe SMO for this purpose. In order to amplify the effects of the drug, SMO can also be used in combination with another anti-craving drug (such as NTX) or adversive drug (such as DF). It seems that when SMO is combined with other drugs with different mechanisms of actions, they work without suppressing the favorable effects of each other.

In conclusion, we believe that SMO can be considered one of the most effective drugs in the treatment of AUD, with a good profile in safety [3,11]. However, all patients taking SMO should be closely monitored by specialists working in an Alcohol Addiction Center with the support of a family member in the administration of the drug.

## References

[1] Snead O.C., Gibson K.M. (2005). Gamma-hydroxybutyric acid. N. Engl. J. Med..

[2] Busardò F.P., Jones A.W. (2015). GHB pharmacology and toxicology: Acute intoxication, concentrations in blood and urine in forensic cases and treatment of the withdrawal syndrome. Curr. Neuropharmacol..

[3] Keating G.M. (2014). Sodium oxybate: A review of its use in alcohol withdrawal syndrome and in the maintenance of abstinence in alcohol dependence. Clin. Drug Investig..

[4] Carter L.P., Koek W., France C.P. (2009). Behavioural analysis of GHB: Receptor mechanisms. Pharmacol. Ther..

[5] Palatini P., Tedeschi L., Frison G., Padrini R., Zordan R., Orlando R., Gallimberti L., Gessa G.L., Ferrara S.D. (1993). Dose-dependent absorption and elimination of gamma-hydroxybutyric acid in healthy volunteers. Eur. J. Clin. Pharmacol..

[6] Gessa G.L., Agabio R., Carai M., Lobina C., Pani M.L., Reali R., Colombo G. (2000). Mechanism of the anti-alcohol effect of gamma hydroxybutyric acid (GHB). Alcohol.

[7] Mirijello A., Caputo F., Vassallo G., Rolland B., Tarli C., Gasbarrini A., Addolorato G. (2015). GABAB agonists for the treatment of alcohol use disorder. Curr. Pharm. Des..

[8] Pardi D., Black J. (2006). Gamma-Hydroxybutyrate/sodium oxybate: Neurobiology, and impact on sleep and wakefulness. CNS Drugs.

[9] Alshaikh M.K., Tricco A.C., Tashkandi M., Mamdani M., Straus S.E., BaHammam A.S. (2012). Sodium oxybate for narcolepsy and cataplexy: Systematic review and meta-analysis. J. Clin. Sleep Med..

[10] Mirijello A., D’Angelo C., Ferrulli A., Vassallo G., Antonelli M., Caputo F., Leggio L., Gasbarrini A., Addolorato G. (2015). Identification and management of alcohol withdrawal syndrome. Drugs.

[11] Skala K., Caputo F., Mirijello A., Vassallo G., Antonelli M., Ferrulli A., Walter H., Lesh O., Addolorato G. (2014). Sodium oxybate in the treatment of alcohol dependence: From the alcohol withdrawal syndrome to the alcohol relapse prevention. Expert Opin. Pharmacother..

[12] Leone M.A., Vigna-Taglianti F., Avanzi G., Brambilla R., Faggiano F. (2010). Gamma-hydroxybutyrate (GHB) for treatment of alcohol withdrawal and prevention of relapses. Cochrane Database Syst. Rev..

[13] Caputo F., Skala K., Mirijello A., Ferrulli A., Walter H., Lesh O., Addolorato G. (2014). Sodium oxybate in the treatment of alcohol withdrawal syndrome: A randomized double-blind comparative study *versus* oxazepam. The GATE 1 Trial. CNS Drugs.

[14] Caputo F., Mirijello A., Cibin M., Mosti A., Ceccanti M., Domenicali M., Bernardi M., Maremmani I., Addolorato G. (2015). Novel strategies to treat alcohol dependence with sodium oxybate according to clinical practice. Eur. Rev. Med. Pharmacol. Sci..

[15] Caputo F., Vignoli T., Grignaschi A., Cibin M., Addolorato G., Bernardi M. (2014). Pharmacological management of alcohol dependence: From mono-therapy to pharmacogenetics and beyond. Eur. Neuropsychopharmacol..

[16] Wang Y.G., Swick T.J., Carter L.P., Thorpy M.J., Benowitz N.L. (2011). Sodium oxybate: Updates and correction to previously published safety data. J. Clin. Sleep Med..

[17] Pross N., Patat A., Vivet P., Bidaut M., Fauchoux N. (2015). Pharmacodynamic interactions of a solid formulation of sodium oxybate and ethanol in healthy volunteers. Br. J. Clin. Pharmacol..

[18] Caputo F., Addolorato G., Stoppo M., Francini S., Vignoli T., Lorenzini F., del Re A., Comaschi C., Andreone P., Trevisani F. (2007). Comparing and combining gamma-hydroxybutyric acid (GHB) and naltrexone in maintaining abstinence from alcohol: An open randomised comparative study. Eur. Neuropsychopharmacol..

[19] Maremmani A.G.I., Pani P.P., Rovai L., Pacini M., Dell’Osso L., Maremmani I. (2011). Long-term γ-Hydroxybutyric acid (GHB) and disulfiram combination therapy in GHB treatment-resistant chronic alcoholics. Int. J. Environ. Res. Public Health.

[20] Connor J.P., Haber P.S., Hall W.D. (2015). Alcohol use disorders. Lancet.

[21] Rehm J., Baliunas D., Borges G.L.G., Graham K., Irving H., Kehoe T., Parry C.D., Patra J., Popova S., Poznyak V. (2010). The relation between different dimensions of alcohol consumption and burden of disease: An overview. Addiction.

[22] Testino G., Leone S., Borro P. (2014). Treatment of alcohol dependence: Recent progress and reduction of consumption. Minerva Med..

[23] Gastfriend D.R., Garbutt J.C., Pettinati H.M., Forman R.F. (2007). Reduction in heavy drinking as a treatment outcome in alcohol dependence. J. Subst. Abus. Treat..

[24] Van Amsterdam J., van den Brink W. (2013). Reduced-risk drinking as a viable treatment goal in problematic alcohol use and alcohol dependence. J. Psychopharmacol..

[25] Gallimberti L., Ferri M., Ferrara S.D., Fadda F., Gessa G.L. (1992). Gamma-hydroxybutyric acid in the treatment of alcohol dependance: A double-blind study. Alcohol. Clin. Exp. Res..

[26] Addolorato G., Cibin M., Caputo F., Capristo E., Gessa G.L., Stefanini G.F., Gasbarrini G. (1998). Gamma-hydroxybutyric acid in the treatment of alcoholism: Dosage fractioning utility in non-responder alcoholic patients. Drug Alcohol Depend..

[27] Addolorato G., Castelli E., Stefanini G.F., Casella G., Caputo F., Marsigli L., Bernardi M., Gasbarrini G. (1996). An open multicentric study evaluating 4-hydroxybutyric acid sodium salt in the medium-term treatment of 179 alcohol dependent subjects. GHB Study Group. Alcohol Alcohol..

[28] Maremmani I., la Manna F., Tagliamonte A. (2001). Long-term therapy using GHB (sodium gamma hydroxybutyrate) for treatment-resistant chronic alcoholics. J. Psychoact. Drugs.

[29] Caputo F., Francini S., Brambilla R., Vigna-Taglianti F., Stoppo M., del Re A., Leggio L., Addolorato G., Zoli G., Bernardi M. (2011). Sodium oxybate in maintaining alcohol abstinence in alcoholic patients with and without psychiatric comorbidity. Eur. Neuropsychopharmacol..

[30] Caputo F., del Re A., Brambilla R., Grignaschi A., Vignoli T., Vigna-Taglianti F., Addolorato G., Zoli G., Cibin M., Bernardi M. (2014). Sodium oxybate in maintaining alcohol abstinence in alcoholic patients according to Lesch typologies: A pilot study. J. Psychopharmacol..

[31] Zvosec D.L., Smith S.W., Quinn Strobl T.P.A., Dyer J.E. (2011). Case series of 226 g-hydroxybutyrate–associated deaths: Lethal toxicity and trauma. Am. J. Emerg. Med..

[32] Martinotti G., Lupi M., Carlucci L., Cinosi E., Santacroce R., Acciavatti T., Chillemi E., Bonifaci L., Janiri L., Di Giannantonio M. (2015). Novel psychoactive substances: Use and knowledge among adolescents and young adults in urban and rural areas. Hum. Psychopharmacol. Clin. Exp..

[33] Vento A.E., Martinotti G., Cinosi E., Lupi M., Acciavatti T., Carrus D., Santacroce R., Chillemi E., Bonifaci L., di Giannantonio M. (2014). Substance use in the club scene of Rome: A pilot study. BioMed. Res. Int..

[34] Degenhardt L., Darke S., Dillon P. (2002). GHB use among Australians: Characteristics, use patterns and associated harm. Drug Alcohol Depend..

[35] Schulz M., Iwersen-Bergmann S., Andresen H., Schmoldt A. (2012). Therapeutic and toxic blood concentrations of nearly 1,000 drugs and other xenobiotics. Crit. Care.

[36] Thai D., Dyer J.E., Jacob P., Haller C.A. (2007). Clinical pharmacology of 1,4-butanediol and gamma-hydroxybutyrate after oral 1,4-butanediol administration to healthy volunteers. Clin. Pharmacol. Ther..

[37] Couper F.J., Logan B.K. (2001). GHB and driving impairment. J. Forensic Sci..

[38] Fieier E.L., Coleman D.E., Baselt R.C. (1998). Gamma-hydroxybutyrate concentrations in pre- and postmortem blood and urine. Clin. Chem..

[39] Elliott S.P. (2004). Further evidence for the presence of GHB in postmortem biological fluid: Implications for the interpretation offindings. J. Anal. Toxicol..

[40] Ferrara S.D., Tedeschi L., Frison G., Rossi A. (1995). Fatality due to gamma-hydroxybutyric acid (GHB) and heroin intoxication. J. Forensic Sci..

